# No effect of repeated post-resistance exercise cold or hot water immersion on in-season body composition and performance responses in academy rugby players: a randomised controlled cross-over design

**DOI:** 10.1007/s00421-022-05075-2

**Published:** 2022-10-25

**Authors:** Barry G. Horgan, Shona L. Halson, Eric J. Drinkwater, Nicholas P. West, Nicolin Tee, Rebekah D. Alcock, Dale W. Chapman, G. Gregory Haff

**Affiliations:** 1 Australian Institute of Sport (AIS), Australian Sports Commission, Bruce, ACT Australia; 2 School of Medical and Health Sciences, Human Performance Centre, Edith Cowan University (ECU), Joondalup, WA Australia; 3 Brumbies Rugby, Bruce, ACT Australia; 4 Australian Catholic University, Banyo, QLD Australia; 5 Centre for Sport Research, School of Exercise & Nutrition Sciences, Deakin University, Geelong, VIC Australia; 6 School of Medical Science and Menzies Health Institute QLD, Griffith University, Brisbane, QLD Australia; 7 Australian Catholic University, Watson, ACT 2602 Australia; 8 Melbourne Football Club, Melbourne, VIC Australia; 9 Curtin University, Bentley, WA 6102 Australia; 10 Directorate of Psychology and Sport, University of Salford, Salford, Greater Manchester UK

**Keywords:** Strength training, Team sports, Hydrotherapy, Recovery, Anthropometry

## Abstract

**Purpose:**

Following resistance exercise, uncertainty exists as to whether the regular application of cold water immersion attenuates lean muscle mass increases in athletes. The effects of repeated post-resistance exercise cold versus hot water immersion on body composition and neuromuscular jump performance responses in athletes were investigated.

**Methods:**

Male, academy Super Rugby players (*n* = 18, 19.9 ± 1.5 y, 1.85 ± 0.06 m, 98.3 ± 10.7 kg) participated in a 12-week (4-week × 3-intervention, i.e., control [CON], cold [CWI] or hot [HWI] water immersion) resistance exercise programme, utilising a randomised cross-over pre–post-design. Body composition measures were collected using dual-energy X-ray absorptiometry prior to commencement and every fourth week thereafter. Neuromuscular squat (SJ) and counter-movement jump (CMJ) performance were measured weekly. Linear mixed-effects models were used to analyse main (treatment, time) and interaction effects.

**Results:**

There were no changes in lean (*p* = 0.960) nor fat mass (*p* = 0.801) between interventions. CON (*p* = 0.004) and CWI (*p* = 0.003) increased (*g* = 0.08–0.19) SJ height, compared to HWI. There were no changes in CMJ height (*p* = 0.482) between interventions.

**Conclusion:**

Repeated post-resistance exercise whole-body CWI or HWI does not attenuate (nor promote) increases in lean muscle mass in athletes. Post-resistance exercise CON or CWI results in trivial increases in SJ height, compared to HWI. During an in-season competition phase, our data support the continued use of post-resistance exercise whole-body CWI by athletes as a recovery strategy which does not attenuate body composition increases in lean muscle mass, while promoting trivial increases in neuromuscular concentric-only squat jump performance.

## Introduction

Resistance exercise is used by athletes to induce muscle hypertrophy and neuromuscular performance enhancement, which underpins maximal strength, power, and speed development. Post-resistance exercise recovery strategies, such as water immersion, are used to expedite recovery and optimise neuromuscular performance. The use of whole-body, i.e., to the neck (Jungmann et al. 2018), water immersion may increase the time available for recovery and adaptation to occur prior to subsequent same- or next-day training sessions. Recovery is believed to be enhanced via the hydrostatic pressure and temperature effects of water immersion which alter the acute post-resistance exercise physiological response (Petersen and Fyfe 2021). For highly trained athlete cohorts, there is a lack of evidence on the benefits or otherwise of repeatedly utilising post-resistance exercise water immersion for muscular hypertrophy and neuromuscular performance (Fyfe et al. 2019). Many studies utilise ‘recreationally active’ subjects, from which results cannot be directly translated to athletes. Furthermore, studies utilising highly trained athletes apply cold water immersion (CWI) following sport-specific training and not following resistance exercise (Ihsan et al. 2021). This makes it difficult to determine the impact of the repeated use of post-resistance exercise water immersion on muscular hypertrophy and neuromuscular performance responses in athletes over time.

In non-athlete participants, the repeated use of post-resistance exercise CWI has been shown to attenuate chronic responses in lean muscle mass (Roberts et al. 2015) or muscle thickness (Poppendieck et al. 2020), which may have contributed to attenuated increases in strength and power performance responses (Poppendieck et al. 2020). If CWI is used repeatedly following resistance exercise, this may result in the repetitive accrual of acute reductions in myofibrillar protein synthesis (Fuchs et al. 2020a,b) and down-regulation of satellite cell activity and hypertrophy signalling pathways (Roberts et al. 2015). This has contributed to scepticism regarding repeated use of CWI application following resistance exercise. However, it is not understood how these findings apply to highly trained athletes who are exposed to increased training and competition demands. In terms of neuromuscular performance, it has been demonstrated that post-exercise CWI helps athletes to accrue increased gym-based volume load, i.e., mean load lifted per set, in subsequent sessions (Roberts et al. 2014), which has been shown to stimulate hypertrophy and neuromuscular strength performance in untrained subjects (Schoenfeld et al. 2016).

Conversely, hot water immersion (HWI) offers practitioners an alternative post-exercise strategy following resistance exercise to promote positive adaptive responses. Physiologically, HWI increases peripheral muscular blood flow, as well as skin, muscle, and core temperature (Bonde-Petersen et al. 1992), resulting in no effect (Stevens et al. 2020) or improved performance in humans (Jackman 2019; Méline et al. 2021), and increased hypertrophy as demonstrated in animal (McGorm et al. 2018) and human models (Rodrigues et al. 2020a,b). In unpublished doctoral thesis work, McGorm (2019) reported that the application of post-exercise HWI (15-min at 45 °C) attenuated increases in lower body muscle mass in recreationally active subjects following a 10-week muscle hypertrophy and strength programme, compared to control (CON) (McGorm 2019). As highly trained athletes are reported to immerse themselves in hot water temperatures of ~ 38–42 °C (Rodrigues et al. 2020a, b; Méline et al. 2021), it remains unclear if this strategy will optimally stimulate muscular adaptation (Fuchs et al. 2020a, b; Stevens et al. 2020) and neuromuscular performance responses (Stevens et al. 2020).

To date, researchers have not investigated the effects of repeated use of post-resistance exercise water immersion on muscular hypertrophy and neuromuscular performance responses in a highly trained athlete cohort (Roberts et al. 2015). This study aimed to determine the effects following a repeated, i.e., 4-week, CWI (15-min × 15 ºC) versus HWI (15-min × 39 ºC) strategy replicating pool temperatures and durations typically utilised by athletes in a high-performance training centre, compared to CON, on the muscular hypertrophy and neuromuscular performance responses following resistance exercise. We hypothesised that neuromuscular performance would increase following repeated bouts of post-resistance CWI, and decrease following repeated bouts of post-resistance HWI. Furthermore, we hypothesised that the chronic lean muscle mass response would decrease following CWI and increase following HWI.

## Methods

### Subjects

Using a repeated-measure study design, with conservative inputs of an effect size *f *= 0.15, alpha = 0.1, 3 groups (CWI, HWI, CON) with an estimated total sample of 24 participants with 3 measures (time) and a correlation among repeated measures of 0.85, the achieved beta (power) is 0.823 (G*Power 3.1.9.2 software). This estimated sample accounts for additional participants to improve the confidence of correlations and to account for the risk of participant drop-out. Thirty-one sub-elite, male, academy Super Rugby players participated. At the point of study commencement, rugby pre-season was fully completed by all subjects, who were now participating in their club adult rugby in-season competition phase, and volunteered to participate in this study as part of their Super Rugby academy membership. Study participation required that all subjects were accustomed to a minimum twice weekly resistance training for > 12 months prior to study commencement. Players had typically completed a minimum of 2-year supervised strength and conditioning sessions prior to participating in this Super Rugby academy pathway tier. This study was scheduled during an in-season phase to facilitate the 12-week intervention given the pre-season phase is not typically of sufficient length. Study approval was obtained from Edith Cowan University and the Australian Institute of Sport (AIS) Human Research Ethics Committees (Approval#:17049). Clinical trial registration was obtained (ANZCTR#:12617000366358), with procedures adhering to the Declaration of Helsinki (2013). Subjects were screened for health and cardiovascular risk using a pre-exercise questionnaire (Adult Pre-exercise Screening System, ESSA, Australia). Subjects were also surveyed regarding any medication or nutritional supplements they were consuming by a qualified sports dietician, prior to confirming suitability to participate in the study. Subjects were informed of study risks and requirements prior to volunteering written informed consent. Prior to study commencement, subjects were familiarised with resistance exercise procedures, post-exercise treatment interventions, and measurement test protocols. Subjects were required to complete a minimum of 87.5% (i.e., 7 of 8) of resistance exercise sessions and post-exercise intervention strategies to meet the inclusion criteria for each 4-week block. Subjects were excluded due to injuries sustained during their club rugby training or games. Data for 18 subjects (*n *= 18, age: 19.9 ± 1.5 y, height: 1.85 ± 0.06 m, weight: 98.3 ± 10.7 kg, body-fat: 18.7 ± 4.1%, body-mass index: 28.4 ± 2.5 kg·m^2^, squat jump: 43.1 ± 7.5 cm, counter-movement jump: 45.4 ± 8.4 cm; Yo–Yo Intermittent Recovery Test Level 1: 1560 ± 440 m) were included in the analysis, distributed across CON (*n* = 17), CWI (*n* = 17) and HWI (*n* = 15) interventions.

### Study design

**Fig. 1:**
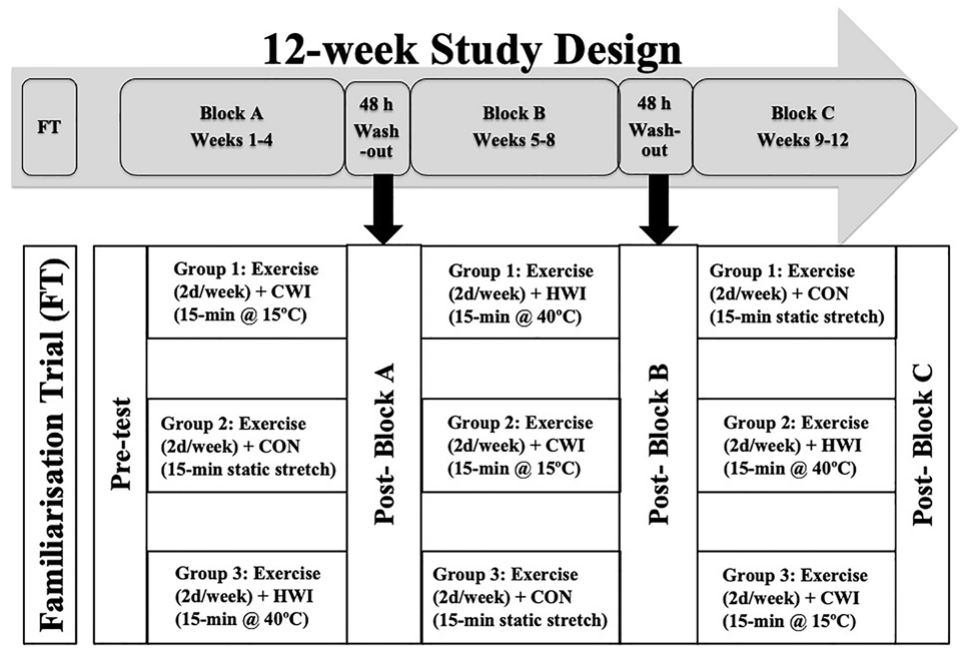
Study design overview

**Fig. 2:**
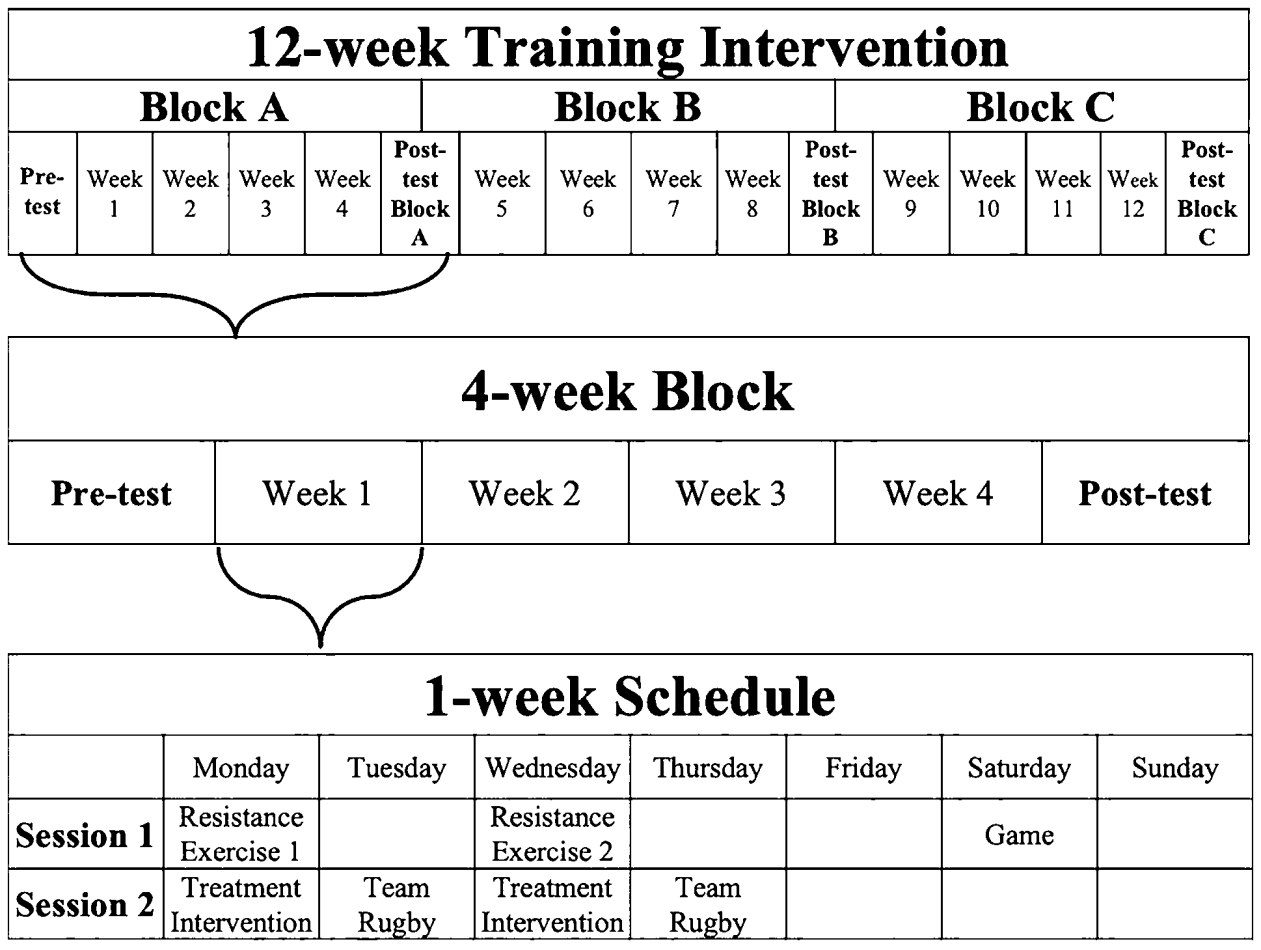
Training intervention schedule overview. Resistance exercise day 1 (Table 1), and day 2 (Table 2), was performed each Monday and Wednesday, respectively, prior to the post-exercise treatment intervention strategy (i.e., CON, CWI or HWI)

A randomised controlled cross-over pre–post-design (Fig. 1) was used, in which subjects undertook a 12-week (4-week $\times$ 3-intervention, i.e., CON, CWI, HWI) programme (Table 1–2) during a club rugby in-season competition phase. A total of eight, i.e., two per week over a 4-week period, resistance exercise sessions occurred immediately prior to one of the three post-resistance exercise treatment interventions, i.e., CON, CWI or HWI, which remained consistent for the duration of each 4-week block (Fig. 2). Resistance exercise was individualised using percentage of body-mass, exercise load-movement velocity, or one repetition maximum. All resistance exercise sessions commenced at the same time of day, i.e., evening, were supervised and performed at room temperature (23–25 °C), with all treatment interventions commencing 15-min post-exercise. Training load was monitored using session rating of perceived exertion (sRPE) (McGuigan and Foster 2004). Subjects consumed whey protein isolate (BodyScience, Australia) post-resistance exercise and prior to commencing the treatment intervention, providing 25 g of protein, 3.2 g of leucine, and 5.8 g of branched-chain amino acids. Subjects remained in the same post-exercise intervention strategy, i.e., 15-min CON, CWI (15.1 ± 0.1 °C) or HWI (39.3 ± 0.6 °C) for each 4-week block and crossed-over thereafter. The CON condition performed static stretching at room temperature (23.0 ± 0.6 °C). The pool water temperature, immersion duration, and protocol utilised in this study is consistent with previous studies, (Amir et al. 2016, Horgan et al. 2021) practical recommendations, (Versey et al. 2013) and applied practice in a national high-performance Olympic training centre. Subjects in the water immersion strategies sat in the pool, immersed to the neck. Pool water was circulated, with temperatures monitored using a thermometer (Testo AG, Germany). Subjects were advised to avoid showering or bathing for > 2 h after all treatment conditions. Body composition was measured every fourth week at the start and end of each 4-week treatment intervention period, while neuromuscular performance was measured weekly at the start of resistance exercise sessions.

**Table 1:** Description of resistance exercise session 1 completed prior to each post-exercise intervention strategy

Resistance exercise day 1		Week 1	Week 2	Week 3	Week 4	Rest
A. Warm up. *LB: SL squat, OH lateral leg raise, SL hip extension; UB: YTW's, external shoulder raises, SA push up*	Reps	2 × 10	2 × 10	2 × 10	2 × 10	n/a
	% 1RM	n/a	n/a	n/a	n/a	
B1. BB back squat	Reps	4 × 8	8,8,6,5	6,6,5,3	5,5,3,2	On 4 min turnaround
	% 1RM	66	66,66,72,77	72,72,77,82	77,77,82,87	
B2. Roman chair prone sl isometric hold	Reps	3 × 30 s	3 × 30 s	3 × 30 s	3 × 30 s	
	% 1RM	n/a	n/a	n/a	n/a	
C1. BB rack pull	Reps	3 × 6	3 × 6	3 × 5	3 × 4	On 4 min turnaround
	% 1RM	65	70	75	80	
C2. Nordic curl	Reps	3 × 4	3 × 4	3 × 4	3 × 4	
	% 1RM	n/a	n/a	n/a	n/a	
D1. BB bench press	Reps	4 × 8	8,8,6,5	6,6,5,3	5,5,3,2	On 4 min turnaround
	% 1RM	66	66,66,72,77	72,72,77,82	77,77,82,87	
D2. Pull up (prone grip)	Reps	4 × 8	8,8,6,5	6,6,5,3	5,5,3,2	
	% 1RM	66	66,66,72,77	72,72,77,82	77,77,82,87	
E1. DB SA shoulder press	Reps	3 × 10	3 × 8	3 × 6	3 × 5	On 4 min turnaround
	% 1RM	65	70	75	80	
E2. SA DB row	Reps	3 × 10	3 × 8	3 × 6	3 × 5	
	% 1RM	65	70	75	80	
F. Core*. 60 s front plank, 30 s es side bridge, 60 s prone bridge*	Reps	3 sets each	3 sets each	3 sets each	3 sets each	15 s between sets
	% 1RM	n/a	n/a	n/a	n/a	

*LB* lower body, *UB* upper body, *OH* overhead, *SA* single-arm, *SL* single-leg, *Reps* repetitions, %*1RM*: percent of 1 repetition maximum, *BB* barbell, *DB* dumbbell, *s* seconds, *es*: each side, *min* minute, *n/a* non-applicable, 2 × 10: Indicates 2 sets × 10 repetitions were completed; 8, 8, 6, 5: indicates that a set of 8, 8, 6 and 5 repetitions were performed, i.e., a total of 4 sets

**Table 2:** Description of resistance exercise session 2 completed prior to each post-exercise intervention strategy

Resistance exercise day 2		Week 1	Week 2	Week 3	Week 4	Rest
A. Warm up *LB: SL squat, OH lateral leg raise, SL hip extension;* *UB: YTW's, external shoulder raise and prone press*	Reps	2 × 10	2 × 10	2 × 10	2 × 10	n/a
	% 1RM	n/a	n/a	n/a	n/a	
B. 15-min run drilling and accelerations (15-min). *60 s skipping, A-march, A-skip, A-drill, accelerations*	Reps	3 × 20 m	3 × 20 m	3 × 20 m	3 × 20 m	On 4 min turnaround
	% 1RM	n/a	n/a	n/a	n/a	
C1. Jump monitoring (SJ, and CMJ)	Reps	2 × 5	2 × 5	2 × 5	2 × 5	
	% 1RM	n/a	n/a	n/a	n/a	
C2. BB jump squat	Reps	4 × 5	4 × 5	5,5,4,3	4,4,3,2	On 4 min turnaround
	% 1RM	65	70	70,70,75,80	75,75,80,85	
D1. BB power shrug (floor)	Reps	4 × 5	4 × 5	5,5,4,3	4,4,3,2	
	% 1RM	65	70	70,70,75,80	75,75,80,85	
D2. BB 2-step step up to hip-lock	Reps	4 × 4 es	4 × 4 es	4 × 4 es	4 × 4 es	On 4 min turnaround
	% 1RM	30 kg	30 kg	30 kg	30 kg	
E1. 3-way DB shoulder raise	Reps	3 × 6	3 × 5	3 × 4	3 × 3	
	% 1RM	65	70	75	80	
E2. DB alternating bench press	Reps	3 × 10	3 × 8	3 × 6	3 × 5	On 4 min turnaround
	% 1RM	65	70	75	80	
E3. Seated row	Reps	3 × 10	3 × 8	3 × 6	3 × 5	
	% 1RM	65	70	75	80	
F. Core. *12* × *hanging leg raise, 20* × *BB roll-out, 10 es x lateral pallof press*	Reps	3 sets each	3 sets each	3 sets each	3 sets each	15 s between sets
	% 1RM	n/a	n/a	n/a	n/a	

*LB* lower body, *UB* upper body, *OH* overhead, *SA* single-arm, *SL* single-leg, *Reps* repetitions, % 1*RM* percent of 1 repetition maximum, *BB* barbell, *DB* dumbbell, *es* each side, *s* seconds, min: minute, *n/a* non-applicable, 2 × 10: Indicates 2 sets × 10 repetitions were completed; 5, 5, 4, 3: indicates that a set of 5, 5, 4, and 3 repetitions were performed, i.e., a total of 4 sets

### Body composition

Measurement of body composition occurred following a rest day, immediately prior to week 1 (pre-test), and every fourth week thereafter at the end of each 4-week intervention, i.e., at the end of weeks 4, 8 and 12. Body composition testing always occurred fasted, in the morning prior to the subjects’ weekly competitive game (Fig. 2). Dual-Energy X-Ray Absorptiometry (DXA) (enCORE v16, Lunar iDXA, General Electric Company, UK) was used to assess body composition, in accordance with AIS best practice protocols. Trained DXA technicians were blinded to participant treatment interventions. Subjects provided a first void, mid-stream urine sample to assess and control for hydration status (PEN-PRO, USA).

### Performance

Weekly monitoring of lower body neuromuscular performance was measured during resistance exercise session 2 (Table 2) using dynamic tests, i.e., one set of five squat jump (SJ) repetitions, where each repetition commenced in an isometric half squat position held for 3 s prior to jumping, and one set of five counter-movement jump (CMJ) repetitions, using a self-selected squat depth. A linear position transducer (GymAware, Australia) was attached to a dowel placed across the shoulders, with the hands positioned just outside shoulder width. Participants were instructed to jump as high as possible on each repetition. Jumps were analysed using the mean of all five reps for each jump type.

### Load monitoring

**Fig. 3:**
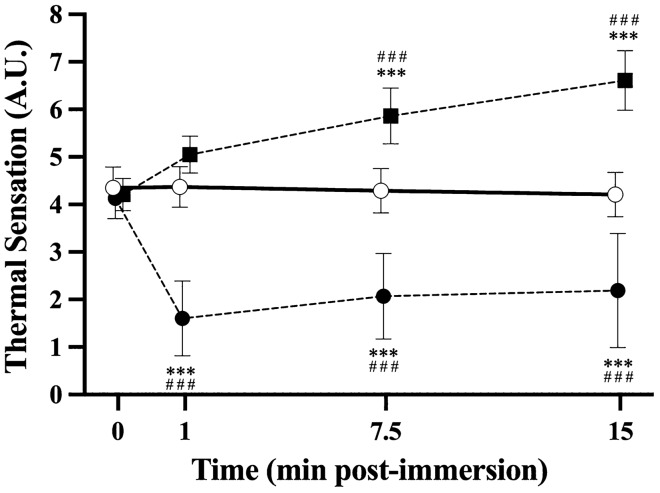
Mean ± SD thermal sensation response over time (min post-immersion) pre- (0 min) and per-immersion (1-, 7.5-, and 15-min post-immersion) intervention strategy following whole-body resistance exercise with either control (CON, *n *= 17), cold (CWI, *n* = 17) or hot (HWI, *n* = 15) interventions. Significant main effects were observed for treatment (*p* < 0.001), time (*p* < 0.001), and treatment × time interaction effect (*p* < 0.001) with Tukey-adjusted *p* values reporting differences for individual treatment × time pair-wise comparisons at 1, 7.5 and 15 min following CWI, and HWI, compared to CON condition; ***: *p* < 0.001 versus CON at pre-immersion baseline; ###: *p* < 0.001 versus CON within time-point

sRPE was surveyed for all team training, competition, and resistance exercise sessions (Table 3). Average sRPE for the weekly resistance exercise sessions 1 and 2 was 5.2 ± 1.3 and 5.1 ± 1.2, respectively, consistent with a subjective rating of “Hard” (Borg 1998). Five-day food diaries were collected. Due to challenges with the accuracy of reporting food diary data (Burke 2015), dietician and statistician advice received advised against analysing the diaries due to missing data-points and risk of bias associated with this data collection method. Thermal sensation (TS) (Fig. 3) was assessed for each post-exercise intervention strategy using a 0–8 Likert scale (0 = “unbearably cold”; 8 = “unbearably hot”) immediately prior (0-min), at one minute (1-min), mid-way (7.5-min) and on completion (15-min) of each strategy.

**Table 3:** Descriptive statistics for measures describing the resistance exercise sessions, total weekly training load, and post-exercise intervention strategies

Training load variable	CON (*N* = 17)	CWI (*N* = 17)	HWI (*N* = 15)
Resistance exercise session 1 sRPE	316.1 ± 79.5	321.5 ± 101.2	345.5 ± 95.9
Resistance exercise session 2 sRPE	327.5 ± 86.5	324.8 ± 107.6	314.9 ± 82.4
Total weekly sRPE	1574.4 ± 523.7	1591.5 ± 607.9	1789.8 ± 528.9

*sRPE* session rating of perceived exertion

### Statistical analyses

Descriptive statistics were calculated prior to analysis using software (Stata/IC 15.1, StataCorp LP, USA). Missing data did not show any obvious pattern and were deemed to be at random and no imputation was made. Data were assessed visually via Q-Q plots, as well as using Shapiro–Wilk’s normality test. Outliers were assessed and identified as ‘severe’ if they were ± 3 SD below or above the 25th or 75th quartiles and the authors searched for any obvious reason to exclude these data. Data were analysed using raw values. Linear mixed-effects models (LMM) were conducted to analyse main (treatment, time) and interaction (treatment × time) effects. Subject were set as a random intercept and the model was fitted via maximum-likelihood estimation using a 1 × 1 covariance matrix structure. The model analysed treatment intervention (i.e., CON, CWI, HWI) and time (group order, i.e., block A, B, C) as fixed effects on the dependent variable, resulting in nine combinations of treatment and time, with two subjects in each such combination. Significance was set at *p* ≤ 0.05. Following LMM, main and interaction effects were evaluated using paired samples t test and corrected using Scheffé’s method of multiple comparison to control for Type 1 error. Intra-class correlations were computed following LMM. Model fit assumptions were assessed via the calculation of residuals. As body composition influences the magnitude of muscle temperature change (Stephens et al. 2017), subject percent of body-fat was used as a fixed effect covariate for response variables. Where significant differences were observed between interventions, practical differences in means were interpreted using Hedges g (1981) effect size (ES) statistic, where g $<$ 0.2 = “trivial”, 0.2 $\le$ g $<$ 0.5 = “small”, 0.50 $\le$ g $<$ 0.8 = “medium”, and g $\ge$ 0.8 = “large”.

## Results

The treatment groups were balanced for total weekly in-season training load across all rugby and resistance exercise sessions (Table 3). The post-exercise water immersion resulted in significant (time, treatment, and time × treatment interaction effects) changes in TS (Fig. 3), compared to CON.

## Body composition measures

**Fig. 4:**
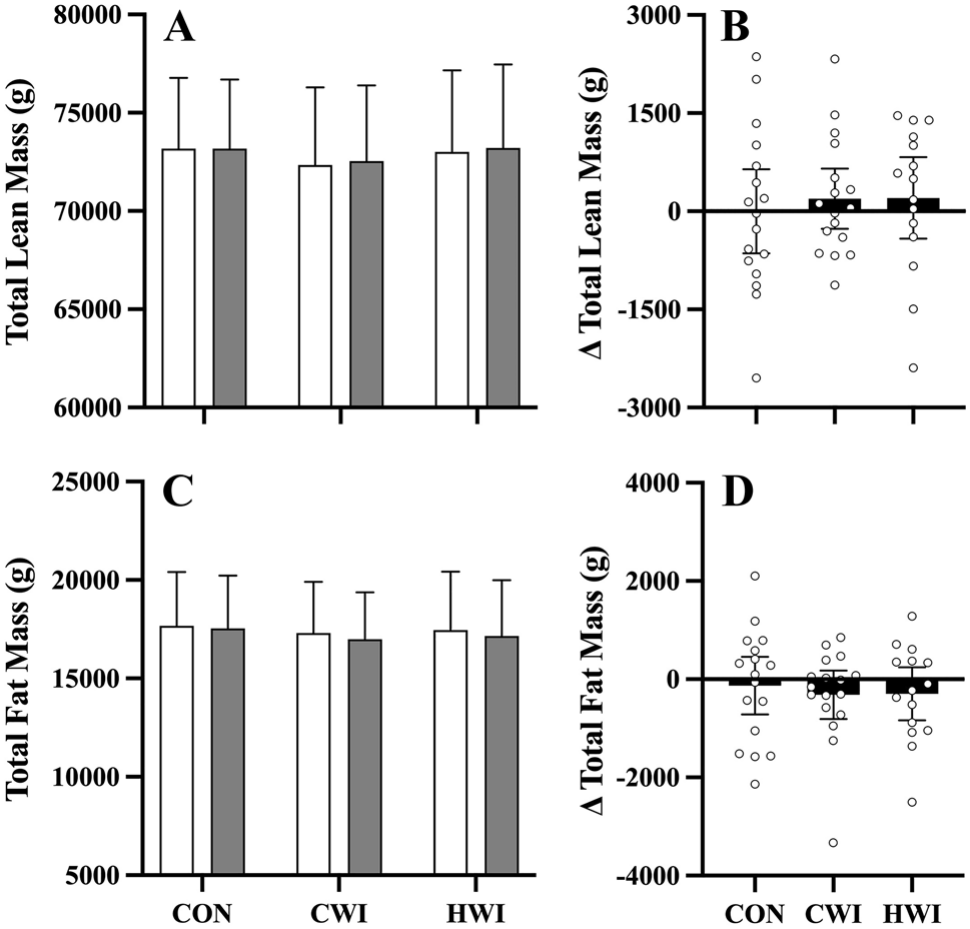
Mean (± 95% C.I.) responses pre- (white bar) versus post-intervention (grey bar), and mean (± 95% C.I.) pre- minus post-intervention delta ($\Delta$) (black bar, and individual data-points) for total lean (panels A–B), and fat (panels C–D) mass following whole-body resistance exercise with either control (CON, *n* = 17), cold (CWI, *n* = 17) or hot (HWI, *n* = 15) water immersion as treatment

Figure 4 shows the effect of post-exercise intervention strategies on body composition changes. Total lean (*p* = 0.960) and fat mass (*p* = 0.801) did not differ significantly between interventions. A significant positive association was observed between total fat mass (*p* < 0.000, *r* = 0.92) and the percent body-fat covariate.

## Performance measures

**Fig. 5:**
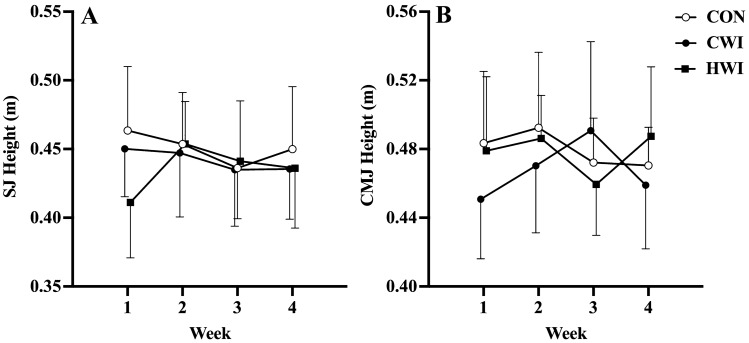
Mean (± 95% C.I.) performance responses across time for SJ (panel A) and CMJ (panel B) height following whole-body resistance exercise with either control (CON, *n* = 17), cold (CWI, *n* = 17) or hot (HWI, *n* = 15) water immersion as treatment. Significant main effects for treatment were observed in SJ height (where CON > HWI, *p* = 0.004, *g* = 0.19; and CWI > HWI, *p* = 0.003, *g* = 0.08); **: *p* ≤ 0.01

Figure 5 shows the effect of post-exercise intervention strategies on performance. CON (*p* = 0.004, *g* = 0.19) and CWI (*p* = 0.003, *g* = 0.08) significantly increased SJ height (Fig. 5, panel A), compared to HWI. Significant negative associations occurred for SJ (*p* = 0.003, *r* = 0.49) and CMJ (*p* = 0.005, *r* = 0.32) height, with the percent body-fat covariate.

## Discussion

During an in-season phase, this study investigated the effects of repeated CWI, HWI or CON on muscular hypertrophy and neuromuscular performance responses following resistance exercise in highly trained athletes. Our study found that repeated post-resistance exercise water immersion (i.e., CWI, HWI) did not affect body composition (i.e., lean nor fat mass) following resistance exercise in this cohort of male, academy Super Rugby players. Post-exercise CWI or CON resulted in significant, trivial increases in SJ height, compared to HWI.

To our knowledge, this is the first study to investigate the effects of repeated post-exercise water immersion on muscle hypertrophy changes in highly trained athletes. Following resistance exercise, we found that repeated CWI or HWI does not influence whole-body lean, nor fat mass. Our results are comparable to research showing no macroscopic (i.e., DXA) change in lean mass for recreationally active subjects (Fyfe et al. 2019). However, the aforementioned study did observe differences between macroscopic (i.e., DXA) and microscopic (i.e., muscle biopsy) morphological measures, where CWI attenuated increases in type II muscle fibre cross-sectional area (Fyfe et al. 2019). Other research utilising non-athlete, physically active, strength trained subjects (Roberts et al. 2015), found repeated CWI attenuated macroscopic (i.e., MRI) increases in muscle cross-sectional area following resistance exercise. Our study, which utilised an all-athlete cohort, supports the theory of potential differences between athletic and recreationally active participants, which may be partly explained by understanding the repetitive accrual of differing muscle protein synthesis processes in trained versus untrained subjects (Damas et al. 2018, Cornish et al. 2020). It is worth emphasising that participants in this study completed post-resistance exercise water immersion treatment during an in-season period where weekly club team rugby training and games were scheduled. The outcomes of this study provide evidence that in-season use of post-resistance exercise water immersion does not interfere with training responses in athletes, as these strategies did not influence body composition changes in lean nor fat mass. Practitioners can prescribe post-exercise water immersion strategies, while simultaneously monitoring individual physiological and performance responses to identify responders versus non-responders. The application of water immersion may contribute to athletes maintaining an ‘adaptive sweet-spot’ under increased training loads, similar to oral anti-inflammatory ingestion following resistance exercise resulting in increased muscular hypertrophy in older adults (Trappe et al. 2010). Considering this alternative hypothesis, further research should investigate the independent and cumulative effects that repeated post-resistance exercise cold versus hot water immersion may have on factors which can influence the adaptive response such as sleep onset, appetite regulation, and subsequent session work-rate and associated training stimuli in athletes.

DXA has been shown to be strongly correlated (*r* = 0.85–0.94) with gold standard lean muscle assessment (e.g., MRI, CT); however, it is understood that DXA is less sensitive (*r* = 0.33 vs. CT) to detect changes in lean muscle mass (Petersen and Fyfe 2021). As such, the use of DXA alone (without simultaneous assessment using CT or MRI) to investigate the macroscopic changes in lean muscle mass may be considered a limitation of this study. Future research utilising athletes should investigate site-specific macroscopic (e.g., MRI, CT, and DXA) changes in lean mass, combined with other non-invasive measures of adaptation, e.g., muscle architecture. A potential limitation of this study which utilised a randomised cross-over design is the short, i.e., 48 h wash-out applied between each 4-week training and treatment block, i.e., blocks A, B and C (Fig. 1). There is a small risk that future adaptations may have occurred from prior completion of the resistance exercise and post-resistance exercise treatment interventions. Future similar study designs should implement a longer wash-out period, in line with expected adaptive responses for the level of athlete participating in the study. Similarly, longer wash-out periods are conducive to implementing pre- and post-testing either side of the wash-out period, and between cross-over to the next treatment block. Additionally, in line with an increased training status associated with an elite athlete cohort, longer duration of training interventions is required to investigate strength and power performance adaptation in response to post-resistance exercise water immersion in athletes (Petersen and Fyfe 2021). As such, future studies should carry out longer duration training interventions when investigating adaptive responses in high-performance, trained athlete cohorts.

In the context of neuromuscular performance, our study observed SJ height demonstrated significant, trivial increases following repeated CWI or CON application after resistance exercise, compared to HWI. Our results are comparable to a study utilising athletes, which reported that chronic post-exercise CWI resulted in small improvements in jump performance in elite Super Rugby players (Tavares et al. 2018), which was similar to recreationally active males (Fyfe et al. 2019). Our results are also comparable to outcomes in short-track speed skaters, where no change occurred in jump performance following repeated post-exercise HWI (Méline et al. 2021). A novel aspect of our study was the comparison of CWI versus HWI. Our findings suggest repeated post-exercise CWI or CON result in trivial increases in in-season concentric jump performance. However, practitioners should be cognisant of individual responses, i.e., responders versus non-responders, as evident by the variation in muscle hypertrophy (Fig. 4B) and across time in neuromuscular jump performance (Fig. 5). As there were no differences in the training load (i.e., sRPE) between treatment interventions, this study did not find evidence to suggest that post-resistance exercise water immersion (e.g., CWI, HWI) is superior to CON in terms of increasing total training load (i.e., perceived workload) over a 4 week block. Further research utilising athletes should investigate the effect of repeated post-resistance exercise water immersion on muscle contractile properties (e.g., electromyography) following resistance exercise, as well as the week-to-week changes over time. Based on the findings reported by Stephens et al (2017) which utilised percent body-fat as an anthropometric covariate, as it may influence muscle and core temperature during water immersion which will have temporal effects to whole-body performance post-immersion, which is further supported by the negative association we report between percent body-fat and jump performance (Stephens et al. 2017).

## Practical applications

Whole-body water immersion strategies as utilised in this study can be prescribed post-resistance exercise for highly trained athletes during an in-season competition phase, posing little risk of attenuating muscular hypertrophy; with the potential to accrue trivial increases in concentric-only SJ height. Future investigations should determine whether individual athlete belief influences chronic perceptual responses to repeated post-resistance exercise hydrotherapy and neuromuscular performance, and to compare this to individual thermal sensation and thermal comfort.

## Conclusions

This investigation provides insights into muscle hypertrophy and neuromuscular performance responses following post-resistance exercise water immersion in athletes. In the context of recent CWI reviews (Petersen and Fyfe 2021), this study provides evidence that post-resistance exercise water immersion can continue to be recommended for use by athletes during in-season competition phases in high-performance training environments. In highly trained athletes, who perform higher frequencies and intensities of training compared to non-athlete counterparts, sufficient evidence does not exist to exclude post-exercise CWI as an in-season recovery strategy for this cohort. This study provides evidence to inform post-resistance exercise water immersion prescription (i.e., timing, temperature) across a 4-week in-season competition phase for highly trained athletes.

## Abbreviations

CON: Control
CMJ: Counter-movement jump
CWI: Cold water immersion
HWI: Hot water immersion
SJ: Squat jump

## References

[CR1] Amir N, Hashim H, Saha S (2016) The effect of single bout of 15 minutes of 15-degree celsius cold water immersion on delayed-onset muscle soreness indicators. International conference on movement, health and exercise, Springer

[CR2] Bonde-Petersen F, Schultz-Pedersen L, Dragsted N (1992). Peripheral and central blood flow in man during cold, thermoneutral, and hot water immersion. Aviat Space Environ Med.

[CR3] Borg G (1998). Borg’s perceived exertion and pain scales.

[CR4] Burke LM (2015). Dietary assessment methods for the athlete: Pros and cons of different methods. Sports Sci Exc.

[CR5] Cornish SM, Bugera EM, Duhamel TA, Peeler JD, Anderson JE (2020). A focused review of myokines as a potential contributor to muscle hypertrophy from resistance-based exercise. Eur J Appl Physio.

[CR6] Damas F, Libardi CA, Ugrinowitsch C (2018). The development of skeletal muscle hypertrophy through resistance training: the role of muscle damage and muscle protein synthesis. Eur J Appl Physiol.

[CR7] Fuchs CJ, Kouw IW, Churchward-Venne TA, Smeets JS, Senden JM, van Marken Lichtenbelt WD, Verdijk LB, van Loon LJ (2020). Postexercise cooling impairs muscle protein synthesis rates in recreational athletes. J Physiol.

[CR8] Fuchs CJ, Smeets JS, Senden JM, Zorenc AH, Goessens JP, van Marken Lichtenbelt WD, Verdijk LB, van Loon LJ (2020). Hot-water immersion does not increase postprandial muscle protein synthesis rates during recovery from resistance-type exercise in healthy, young males. J Appl Physiol.

[CR9] Fyfe JJ, Broatch JR, Trewin AJ, Hanson ED, Argus CK, Garnham AP, Halson SL, Polman RC, Bishop DJ, Petersen AC (2019). Cold water immersion attenuates anabolic signalling and skeletal muscle fiber hypertrophy, but not strength gain, following whole-body resistance training. J Appl Physiol.

[CR10] Horgan BG, West NP, Tee N, Drinkwater EJ, Halson SL, Vider J, Fonda CJ, Haff GG, Chapman DW (2021) Acute inflammatory, anthropometric, and perceptual (muscle soreness) effects of postresistance exercise water immersion in junior international and subelite male volleyball athletes. J Strength Cond Res 10.1519/JSC.0000000000004122 34537801

[CR11] Ihsan M, Abbiss CR, Allan R (2021). Adaptations to post-exercise cold water immersion: friend, foe, or futile?. Front Sports Active Living.

[CR12] Jackman, J. (2019). Turning up the heat: can post-exercise hot water immersion be used to manipulate acute physiological responses and chronic adaptation following resistance training? , Middlesex University.

[CR13] Jungmann M, Vencatachellum S, Van Ryckeghem D, Vögele C (2018). Effects of cold stimulation on cardiac-vagal activation in healthy participants: randomized controlled trial. JMIR Formative Res.

[CR14] McGorm H (2019). The effects of hot water immersion on recovery, performance and adaptation to resistance exercise.

[CR15] McGorm H, Roberts LA, Coombes JS, Peake JM (2018). Turning up the heat: an evaluation of the evidence for heating to promote exercise recovery, muscle rehabilitation and adaptation. Sports Med.

[CR16] McGuigan MR, Foster C (2004). A new approach to monitoring resistance training. Strength and Conditioning Journal.

[CR17] Méline T, Solsona R, Antonietti J-P, Borrani F, Candau R, Sanchez AM (2021). Influence of post-exercise hot-water therapy on adaptations to training over 4 weeks in elite short-track speed skaters. J Exerc Sci Fit.

[CR18] Petersen AC, Fyfe JJ (2021). Post-exercise cold water immersion effects on physiological adaptations to resistance training and the underlying mechanisms in skeletal muscle: a narrative review. Frontiers in Sports and Active Living.

[CR19] Poppendieck W, Wegmann M, Hecksteden A, Darup A, Schimpchen J, Skorski S, Ferrauti A, Kellmann M, Pfeiffer M, Meyer T (2020). Does Cold-water immersion after strength training attenuate training adaptation?. Inter J Sports Physiol Performance.

[CR20] Roberts LA, Nosaka K, Coombes JS, Peake JM (2014). Cold water immersion enhances recovery of submaximal muscle function after resistance exercise. Am J Physiol Regul Integr Comp Physiol.

[CR21] Roberts LA, Raastad T, Markworth JF, Figueiredo VC, Egner IM, Shield A, Cameron-Smith D, Coombes JS, Peake JM (2015). Post-exercise cold water immersion attenuates acute anabolic signalling and long-term adaptations in muscle to strength training. J Physiol.

[CR22] Rodrigues P, Trajano GS, Wharton L, Minett GM (2020). Effects of passive heating intervention on muscle hypertrophy and neuromuscular function: a preliminary systematic review with meta-analysis. J Therm Biol.

[CR23] Rodrigues P, Trajano GS, Wharton L, Minett GM (2020). Muscle temperature kinetics and thermoregulatory responses to 42° C hot-water immersion in healthy males and females. Eur J Appl Physiol.

[CR24] Schoenfeld BJ, Wilson JM, Lowery RP, Krieger JW (2016). Muscular adaptations in low-versus high-load resistance training: a meta-analysis. Eur J Sport Sci.

[CR25] Stephens JM, Halson SL, Miller J, Slater GJ, Chapman DW, Askew CD (2017). Effect of body composition on physiological responses to cold water immersion and the recovery of exercise performance. Int J Sports Physiol Perform.

[CR26] Stevens CJ, Ross ML, Carr AJ, Vallance B, Best R, Urwin C, Périard JD, Burke L (2020). Postexercise hot-water immersion does not further enhance heat adaptation or performance in endurance athletes training in a hot environment. Int J Sports Physiol Perform.

[CR27] Tavares, F., M. Beaven, J. Teles, D. Baker, P. Healey, T. B. Smith and M. Driller (2018). “The effects of chronic cold water immersion in elite rugby players.” International journal of sports physiology and performance: 1–23. 10.1123/ijspp.2018-0313 29952675

[CR28] Trappe TA, Carroll CC, Dickinson JM, LeMoine JK, Haus JM, Sullivan BE, Lee JD, Jemiolo B, Weinheimer EM, Hollon CJ (2010). Influence of acetaminophen and ibuprofen on skeletal muscle adaptations to resistance exercise in older adults. Am J Physiol Regul Integr Comp Physiol.

[CR29] Versey NG, Halson SL, Dawson BT (2013). Water immersion recovery for athletes: effect on exercise performance and practical recommendations. Sports Med.

